# Serial EXTEM, FIBTEM, and tPA Rotational Thromboelastometry Observations in the Maastricht Intensive Care COVID Cohort—Persistence of Hypercoagulability and Hypofibrinolysis Despite Anticoagulation

**DOI:** 10.3389/fcvm.2021.654174

**Published:** 2021-04-26

**Authors:** Anne-Marije Hulshof, Renée A. G. Brüggemann, Mark M. G. Mulder, Tom W. van de Berg, Jan-Willem E. M. Sels, Renske H. Olie, Bart Spaetgens, Alexander S. Streng, Paul Verhezen, Iwan C. C. van der Horst, Hugo Ten Cate, Henri M. H. Spronk, Bas C. T van Bussel, Yvonne M. C. Henskens

**Affiliations:** ^1^Central Diagnostic Laboratory, Maastricht University Medical Centre+, Maastricht, Netherlands; ^2^Cardiovascular Research Institute Maastricht, Maastricht University, Maastricht, Netherlands; ^3^Department of Internal Medicine, Maastricht University Medical Centre+, Maastricht, Netherlands; ^4^Department of Intensive Care Medicine, Maastricht University Medical Centre+, Maastricht, Netherlands; ^5^Thrombosis Expert Centre Maastricht, Maastricht University Medical Centre+, Maastricht, Netherlands; ^6^Care and Public Health Research Institute, Maastricht University, Maastricht, Netherlands

**Keywords:** COVID-19, fibrinolysis, pulmonary embolism, thromboelastometry (ROTEM®), thrombosis

## Abstract

**Background:** Coronavirus Disease 2019 (COVID-19) patients often present with thromboembolic events. In COVID-19 patients, routine hemostatic assays cannot correctly identify patients at risk for thromboembolic events. Viscoelastic testing with rotational thromboelastometry (ROTEM) might improve the characterization of COVID-19-associated coagulopathy.

**Objective:** To unravel underlying coagulopathy and fibrinolysis over time as measured by serial assessment heparin-independent (FIBTEM and EXTEM) and fibrinolysis illustrating (tissue plasminogen activator; tPA) ROTEM assays.

**Patients/Methods:** Between April 23 and June 12, consecutive adult patients enrolled within the Maastricht Intensive Care COVID (MaastrICCht) cohort were included, and a comprehensive set of clinical, physiological, pharmaceutical, and laboratory variables were collected daily. Twice per week, EXTEM, FIBTEM, and tPA ROTEM were performed. Clotting time (CT), clot formation time (CFT), maximum clot firmness (MCF), lysis onset time (LOT), and lysis time (LT) were determined to assess clot development and breakdown and were compared to routine hemostatic assays.

**Results:** In 36 patients, 96 EXTEM/FIBTEM and 87 tPA ROTEM tests were performed during a 6-week follow-up. CT prolongation was present in 54% of EXTEM measurements, which were not matched by prothrombin time (PT) in 37%. Respectively, 81 and 99% of all EXTEM and FIBTEM MCF values were above the reference range, and median MCF remained elevated during follow-up. The ROTEM fibrinolysis parameters remained prolonged with median LOT consequently >49 min and unmeasurable LT in 56% of measurements, suggesting a severe hypofibrinolytic phenotype.

**Conclusion:** ROTEM tests in COVID-19 ICU patients show hypercoagulability and severe hypofibrinolysis persisting over at least 6 weeks.

## Introduction

Early after the onset of the severe acute respiratory syndrome coronavirus 2 (SARS-CoV-2) pandemic, anecdotal reports of COVID-19 patients suffering from extensive thromboembolic complications, mainly pulmonary embolisms, emerged (1, 2). Currently, more extensive studies depicted the incidence of thromboembolic complications in COVID-19 intensive care unit (ICU) patients between 20 and 77%, depending on the screening strategy and reported outcomes (3–12). Additionally, subtraction CT angiography (sCTA) showed abnormally decreased iodine distribution in areas of morphologically normal lung parenchyma of COVID-19 patients (13). These findings indicate a severe ventilation/perfusion mismatch, suggesting a role for the procoagulant state and microthrombi development in disease progression. Evidence indicates that COVID-19-associated coagulopathy plays a pivotal role in morbidity and mortality in critically ill patients (14). However, it is currently unknown whether the observed coagulopathy is directly virus-induced, a response to extensive systemic inflammation, or both. Moreover, a recent study suggests that COVID-19 causes hypercoagulability and leads to a decrease of fibrinolysis, subsequently associated with venous thromboembolism (VTE) (15). Identifying COVID-19 patients at the most significant risk for thromboembolic events is essential to guide optimal anticoagulant prophylaxis and treatment strategies limiting thromboembolic deaths.

Evaluation of COVID-19-associated coagulopathy has proven to be challenging as routine hemostatic assays cannot correctly identify patients at risk for COVID-19-associated thromboembolic complications (16). In part, this may be caused by the fact that multiple systems, including cellular immunity (neutrophils), endothelial cells, complement activation, contact activation, and hypofibrinolysis, are engaged, which mostly remain undetected in routine assays (17, 18). Viscoelastic testing might better represent coagulation *in vivo* than routine hemostatic assays due to its whole blood dynamic assessment. Assessment of the complete fibrinolytic system remains notoriously difficult, but tissue plasminogen activator rotational thromboelastometry (tPA ROTEM) is thought to be more sensitive to hypofibrinolysis than other clinically used assays (19, 20).

We aimed to unravel the underlying coagulopathy as measured with serial heparin-independent EXTEM and FIBTEM, and tPA ROTEM tests in a mechanically ventilated COVID-19 ICU population over several weeks. The prospective and longitudinal aspect of this study, combined with serial tPA ROTEM measurements, is unprecedented in the current field of hemostatic testing in COVID-19.

## Materials and Methods

The manuscript was written following the STrengthening the Reporting of OBservational studies in Epidemiology (STROBE) guidelines (21).

### MaastrICCht Study

The Maastricht Intensive Care COVID (*MaastrICCht*) cohort study design has been described more extensively elsewhere (22). This prospective cohort study was conducted in patients admitted to the ICU of the Maastricht University Medical Centre+ (MUMC+). The study was designed to foster other datasets and registries according to the FAIR data principle in collaboration (23). The local institutional review board [Medisch Ethische Toetsingscomissie (METC) 2020-1565/ 300523] of the MUMC+ approved the study, based on the reglations of the declaration of Helsinki. The study is registered in the Netherlands Trial Register (registration number NL8613). Patients provided consent for use of the collected data for research purposes.

This study included all participants with respiratory insufficiency requiring mechanical ventilation and at least one real-time polymerase chain reaction (RT-PCR) positive for SARS-CoV-2 RNA and/or a chest CT scan strongly suggestive for SARS-CoV-2 infection, based on a CORADS-score of 4–5 scored by a radiologist (24). Participants were followed until the primary outcome was reached (i.e., either died in the ICU or discharge from the ICU). Every day, a comprehensive and uniform set of clinical, physiological, and laboratory variables was collected, reducing the chance of missing data. In addition, when patients were not available for blood withdrawal or laboratory testing failed, the measurement would be rescheduled for the next blood withdrawal. Computed tomography pulmonary angiography (CTPA) was performed when pulmonary embolism was clinically suspected. Due to logistical reasons, no routine compression ultrasonography for the detection of deep venous thrombosis was performed.

### Anticoagulation

During the COVID-19 pandemic, incident thrombosis frequently appeared, resulting in new thromboprophylaxis recommendations. Consequently, from April 23, 2020, the dosage in the ICU was increased (Nadroparin 5,700, 7,600, and 11,400 IU for, respectively, <70, 70–90, and >90 kg) after the release of a national guidance document (25). Patients who required therapeutic anticoagulants before hospital admission were started on therapeutic low molecular weight heparin (LMWH) upon ICU admission; vitamin K antagonists (VKAs) and direct oral anticoagulants (DOACs) were discontinued since oral medication might not be resorbed optimally in critically ill patients. Patients on extracorporeal membrane oxygenation (ECMO) or continuous renal replacement therapy (CRRT) received unfractionated heparin (UFH), monitored using the aPTT [heparin therapeutic range (HTR) 50–80 s, based on an aPTT ratio of 1.5–2.5] and the anti-Xa (HTR 0.3–0.7 IU/ml) according to local guidelines.

### Hemostasis Subcohort

*N* = 94 patients were enrolled from March 25 until June 12 in the MaastrICCht cohort. Enrollment for the hemostasis subcohort started later, on April 23. To align patients within their severe COVID-19 disease course, measurements were classified per week after intubation. ROTEM measurements were started in newly admitted patients from April 23 onwards. Additionally, patients that were on the ICU on April 23 who were already included in the MaastrICCht cohort were also included in the hemostasis subcohort and ROTEMs were measured. When patients were transferred from another ICU to the MUMC+, the original date of intubation was used. This means that some patients could be included in their first, second, third, or even fourth week after intubation. Thus, timing of inclusion in the hemostasis subcohort varied by design. ROTEM measurements were performed twice weekly if logistically possible during a pandemic situation. ROTEM measurements were taken until discharge, transfer to other care facility, or death.

### Blood Withdrawal and Preparation

Daily blood samples from all patients were collected from an arterial line using 7.2 mg K_2_ EDTA (4.0 ml), serum, and 3.2% (w/v) citrate Vacutainer vacuum blood tubes (Becton Dickinson, Plymouth, UK). Additional 3.2% (w/v) citrate blood tubes were collected twice a week for global hemostasis assays, including ROTEM. Whole blood tests were performed within 4 h after blood withdrawal. Platelet-free plasma (PFP) was obtained using two subsequent centrifugation steps: initial centrifugation of 2,490 *g* for 5 min, followed by 10,000 *g* for 10 min.

### Laboratory Measurements

Complete blood count (leukocyte count, platelet count, hemoglobin, and hematocrit) was performed on a Sysmex XN-9000 analyzer (Sysmex Corporation, Kobe, Hyogo, Japan) using EDTA blood. PT (Dade Innovin; Siemens, Marburg, Germany), aPTT (Dade Actin FSL; Siemens), fibrinogen concentration (Clauss method, Dade Thrombin Reagent; Siemens), D-dimer concentration (Innovance; Siemens), and Anti-Xa activity (Biophen Heparin LRT; Hyphen Biomed, Neuville-Sur-Oise, France) were measured on a Sysmex CS2100i (Sysmex Corporation) in 3.2% citrated blood. For the anti-Xa measurement, COVID-19 patient samples (18 μl) were three times diluted with reference pooled plasma (36 μl). Anti-Xa activity was determined using an LMWH calibration line (aXa-LMWH; Hyphen Biomed). UFH activity was subsequently calculated with a previously determined formula: UFH anti-Xa = 1.55 ^*^ LMWH anti-Xa (26). Bilirubin (Bilirubin Total, third-generation; Roche Diagnostics, Basel, Switzerland) and creatinine (Enzymatic Reagent; Roche Diagnostics) were determined in serum on a COBAS® 8000 (Roche Diagnostics).

### ROTEM and tPA ROTEM

ROTEM (Werfen; Barcelona, Spain) is a point-of-care viscoelastic assay performed in 3.2% (w/v) citrated whole blood. The ROTEM sigma cartridge-based system was used to perform EXTEM and FIBTEM to illustrate clot development's dynamic properties. EXTEM and FIBTEM are activated with tissue factor, with additional cytochalasin D added to the FIBTEM reagent to eliminate platelet contribution to clot formation. FIBTEM highlights the fibrinogen contribution to clot formation, as opposed to EXTEM, which illustrates all components' contribution to clot buildup. Polybrene is present in both assays to inhibit a potential anticoagulant effect of heparin. Standard ROTEM parameters were analyzed: clotting time (CT, in seconds), clot formation time (CFT, in seconds), and maximum clot firmness (MCF, in millimeters).

TPA ROTEM was performed to assess the fibrinolytic properties in COVID-19 patients. Recombinant tissue plasminogen activator (r-tPA) is used to create an EXTEM-like assay, previously validated by Kuiper et al. (20). In brief, 125 ng/ml r-tPA (Actilyse; Boehringer Ingelheim BV) is added in combination with tissue factor (Dade Innovin, Siemens) and calcium to activate clot formation and fibrinolysis simultaneously. ROTEM parameters used to evaluate fibrinolysis are Ly60 (percentage of MCF still present at 60 min), lysis onset time (LOT; time until 15% breakdown of MCF, in minutes), and lysis time (LT; time until 90% breakdown of MCF, in minutes). The tPA assay was performed on a ROTEM delta, enabling manual addition of reagents and lasted for a maximum of 2 h. If LOT and/or LT were not reached within 2 h, these results were capped at 120 min for analysis. For clinical reference, it is important to note that prolonged LOT and LT indicate delayed or absent fibrinolysis, defined in the current manuscript as hypofibrinolysis. Reversely, shorter LOT and LT indicate hyperfibrinolysis.

### Statistical Analyses

Descriptive results were presented per week with the time of intubation as reference. The time between the day of intubation and the first ROTEM test was variable by design, as several patients were transferred from other ICUs and were intubated before MUMC+ ICU admission and ROTEM assays started from April 23 onwards. This allowed us to investigate weekly ROTEM parameters on the cohort level over several weeks, aiming to study patients at a similar disease progression stage. We used only the first measurement per week for each individual patient and studied the first 6 weeks in pooled analyses (i.e., week 1: days 1–7 after intubation; week 2: days 8–14 after intubation; until week 6). However, when we repeated the analyses using the average ROTEM measurements per week results did not materially change (data not shown). Measurements over 6 weeks after intubation (*n* = 11) were excluded from pooled analyses since the number of weekly measurements fell below 10, and no reliable analysis could be performed. A detailed overview of this cohort was provided for more insight as Supplementary Figure 1. Descriptive data are presented as mean (standard deviation; SD) or median [interquartile range; IQR] as appropriate.

Linear regression was performed on all measurements (weeks 1–10). Linear regression analyses were used to evaluate ROTEM parameters' determinants, and presented are the standardized β coefficient with 95% confidence intervals (95%CI) and *R*^2^. A *p*-value of <0.05 was considered statistically significant. Statistical analyses were performed in IBM SPSS Statistics 25.0, and figures were created using GraphPad Prism.

## Results

### Demographic Characteristics

Thirty-six COVID-19 ICU patients were included in the present study. The median age was 61 years (IQR 55–70 years, range 17–75 years), and 81% was male. Baseline characteristics, ICU admission, and events are presented in Table 1. All patients were mechanically ventilated during ICU stay, and few comorbidities were present. Twenty patients (55.6%) had a confirmed pulmonary embolism, and mortality during ICU admission was 19% (*n* = 7).

**Table 1:** Baseline characteristics, ICU admission, and events.

**Parameter**	**Mean (SD) or Median [IQR]** ***N* (%)**
**General**	36 patients
Age (years)	61 [55–70]
Gender (male)	29 (80.6%)
BMI (kg/m^2^)	28 (4.1)
**Medical history**	
Chronic kidney disease	1 (2.8%)
Diabetes mellitus	1 (2.8%)
Hypertension^*^	10 (27.8%)
Malignancy	4 (11.1%)
Myocardial infarction	2 (5.6%)
Peripheral vascular disease	1 (2.8%)
**Medication prior to admission**	
Antiplatelet agent	
• Acetylsalicylic acid	4 (11.1%)
DOAC	
• Apixaban	1 (2.8%)
• Dabigatran	1 (2.8%)
• Rivaroxaban	1 (2.8%)
**ICU admission**	
Admission origin	
• Emergency department	9 (25%)
• Regular ward	14 (38.9%)
• Transfer from other ICU	13 (36.1%)
APACHE II at admission	15 [13–18]
SAPS II at admission	40 [29–44]
**ICU events**	
Length of stay ICU (days)	34 [21–42]
Time on ventilator (days)	27 [17–32]
CRRT during ICU stay (yes)	10 (27.8%)
ECMO during ICU stay (yes)	6 (16.7%)
CT confirmed pulmonary embolism (yes)	20 (55.6%)
Compression ultrasonography confirmed deep venous thrombosis (yes)	1 (2.8%)
ICU mortality (death)	7 (19.4%)

* *Data irretrievable for one patient. BMI, body mass index; CVA, cerebrovascular event; TIA, transient ischemic attack; DOAC, direct oral anticoagulant; ICU, intensive care unit; APACHE, acute physiology and chronic health evaluation; SAPS, simplified acute physiology score; CRRT, continuous renal replacement therapy; ECMO, extracorporeal membrane oxygenation; CT, computed tomography*.

**Figure 1:**
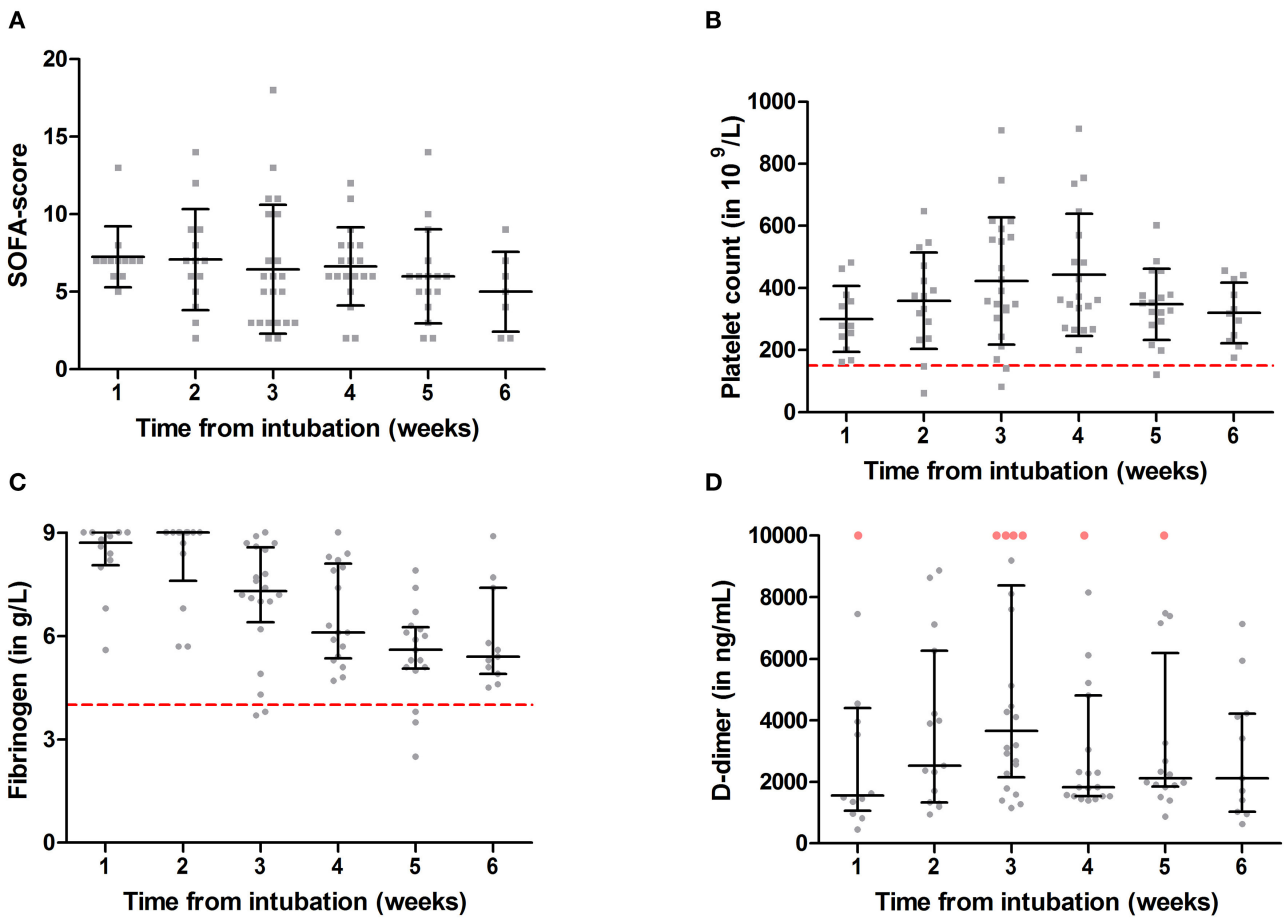
Sequential Organ Failure Assessment (SOFA) score **(A)**, platelet count **(B)**, D-dimer **(C)**, and fibrinogen **(D)** measurements over 6 weeks from intubation in a critically ill COVID-19 population. Results are presented as mean with standard deviation (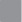) or median with interquartile range (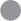); gray symbols illustrate individual measurements. Dashed lines represent reference values for thrombocytopenia (<150 × 10^9^/L) and hyperfibrinogenemia (>4 g/L). D-dimer reference range is age dependent and is defined as <500, <510, <560, <610, <660, and <710 ng/ml for ages <50, 51–55, 56–60, 61–65, 66–70, and 71–75, respectively. Red dots illustrate D-dimer values >10,000 ng/ml.

The median Sequential Organ Failure Assessment (SOFA) score was 7 (IQR 6.5–7) in the first week after intubation (Table 2 and Figure 1A) and remained elevated throughout the 6-week follow-up. The involvement of multi-organ failure during the clinical course of mechanically ventilated patients was shown previously in the MaastrICCht cohort (27). Platelet count was mostly within the normal range (Figure 1B). D-dimer was increased in all patients and did not normalize over time (Figure 1C). Fibrinogen was highly elevated during the first 2 weeks after intubation and slowly declined (Figure 1D), showing a normalization trend during the following weeks. Our laboratory's fibrinogen measurements are capped at 9.0 g/L for routine assays, and no further dilution in fibrinogen samples was performed. Therefore, fibrinogen levels of 9.0 g/L are considered to be even more elevated.

**Table 2:** SOFA score, routine laboratory, standard ROTEM, and tPA ROTEM in COVID-19 patients per week from intubation.

**Parameter**	**Week number**	**1**	**2**	**3**	**4**	**5**	**6**
**ROUTINE LABORATORY**	**Reference range**						
SOFA score		7 (2)	7 (3)	6 (4)	7 (3)	6 (3)	5 (3)
Fibrinogen (g/L)	1.7–4.0	8.7 [8.1–9]	9 [8.4–9]	7.3 [6.6–8.6]	6.1 [5.4–8.0]	5.6 [5.1–6.2]	5.4 [4.9–7.4]
D-dimer (ng/ml)	^*^	1,562 [1,158–4,246]	2,524 [1,339–6,250]	3,652 [2,271–8,108]	1,831 [1,539–4,801]	2,113 [1,857–5,210]	2,118 [1,031–4,222]
PT (s)	9.9–12.4	12.2 (2.0)	12.4 (3.2)	12.4 (2.3)	12.4 (2.3)	12.2 (1.8)	11.3 (0.3)
aPTT (s)	23–32	36 [32–40]	34 [30–52]	31 [29–73]	31 [28–40]	28 [27–30]	29 [25–32]
Platelet count (10^9^/L)	150–350	301 (106)	359 (155)	423 (205)	442 (197)	348 (114)	320 (97)
**STANDARD ROTEM**							
Number of patients		12	15	22	19	17	11
EXTEM CT (s)	49–77	103 [84–129]	103 [79–130]	76 [65–131]	80 [68–86]	78 [70–85]	71 [66–78]
EXTEM CFT (s)	44–129	48 [42–77]	50 [42–65]	44 [37–58]	41 [36–49]	37 [37–43]	38 [36–42]
EXTEM MCF (mm)	55–74	78 (4)	79 (5)	80 (5)	80 (3)	77 (5)	77 (4)
FIBTEM CT (s)	43–100	93 [87–113]	100 [81–145]	77 [68–136]	82 [71–98]	75 [68–87]	74 [68–85]
FIBTEM MCF (mm)	5–19	38 (5)	39 (5)	34 (8)	34 (5)	30 (6)	31 (6)
**TPA ROTEM**							
Number of patients		11	12	22	18	15	9
TPA LY60 (%)		80 [70–91]	75 [45–90]	79 [61–93]	81 [59–93]	82 [57–91]	74 [50–86]
TPA LOT (min)	26–49	55.42 [46.72–80.72]	50.67 [39.00–72.25]	55.51 [47.45–81.90]	56.35 [46.64–120]	53.88 [47.80–77.23]	50.95 [48.56–60.91]
TPA LT (min)	35–77	120 [109.07–120]	108.76 [85.44–120]	120 [85.88–120]	120 [92.74–120]	120 [96.42–120]	96.53 [72.25–114.81]

*Results are presented as mean (standard deviation) or median [interquartile range (IQR)]. Routine laboratory and standard ROTEM reference values are according to MUMC+ guidelines. TPA ROTEM reference values are in accordance with Kuiper et al. (20). Unmeasurable LOT and LT values within 2 h were set at 120 min to calculate adjusted TPA LOT and LT values. SOFA, Sequential Organ Failure Assessment; PT, prothrombin time; CT, clotting time; CFT, clot formation time; MCF, maximum clot firmness; Ly60, Lysis % at 60 min; LOT, lysis onset time; LT, lysis time*.

* *D-dimer reference values are age dependent and defined as <500, <510, <560, <610, <660, and <710 ng/ml for ages <50, 51–55, 56–60, 61–65, 66–70, and 71–75 respectively*.

All patients received some form of anticoagulation during their ICU treatment. Table 3 shows the different types and dosages of anticoagulation and anti-Xa levels.

**Table 3:** Anticoagulation day prior to ROTEM blood withdrawal in COVID-19 patients per week from intubation.

	**Week number**	**1**	**2**	3^*^	**4**	**5**	**6**
**ANTICOAGULATION**							
Number of patients		12	15	22	19	17	11
**UNFRACTIONATED HEPARIN (UFH)**							
UFH use		2 (16.7)	4 (26.7)	9 (40.9)	4 (21.1)	2 (11.8)	0 (0)
UFH dosage (×1,000 IU)		45.2 [–, –]	43.4 [34.5–59.3]	41.1 [31.2–48.0]	41.9 [35.5–44.4]	39.6 [–, –]	–
Anti-Xa (IU/ml)		0.75 (–)	0.61 (0.38)	0.91 (0.51)	0.77 (0.30)	0.53 (–)	–
	Missing data	1	1	1	1	0	–
**LOW-MOLECULAR-WEIGHT HEPARIN (LMWH)**							
LMWH use	Total	10 (83.3)	11 (73.3)	13 (59.1)	14 (73.3)	15 (88.2)	10 (90.9)
	Prophylactic	6 (60.0)	5 (45.5)	6 (46.2)	4 (28.6)	4 (26.7)	3 (30)
	Therapeutic	4 (40.0)	6 (54.5)	7 (53.8)	10 (71.4)	11 (73.3)	7 (70)
Anti-Xa (IU/ml)		0.52 (0.21)	0.49 (0.29)	0.46 (0.23)	0.53 (0.23)	0.57 (0.22)	0.64 (0.25)
	Missing data	0	1	1	2	1	4
**OTHERS**							
Fondaparinux use		–	–	–	–	–	1 (9.1)
No anticoagulation		–	–	1 (4.5)	1 (5.3)	–	–

*Results are presented as n (%) for categorical data and mean (standard deviation) or median [25th percentile−75th percentile] for continuous data. Blood withdrawal for ROTEM measurements was performed from 4:30 to 5:30 a.m.; thus, anticoagulation the day prior to blood withdrawal is presented*.

* *One patient received both UFH and LMWH the day prior to blood withdrawal*.

### ROTEM

**Figure 2:**
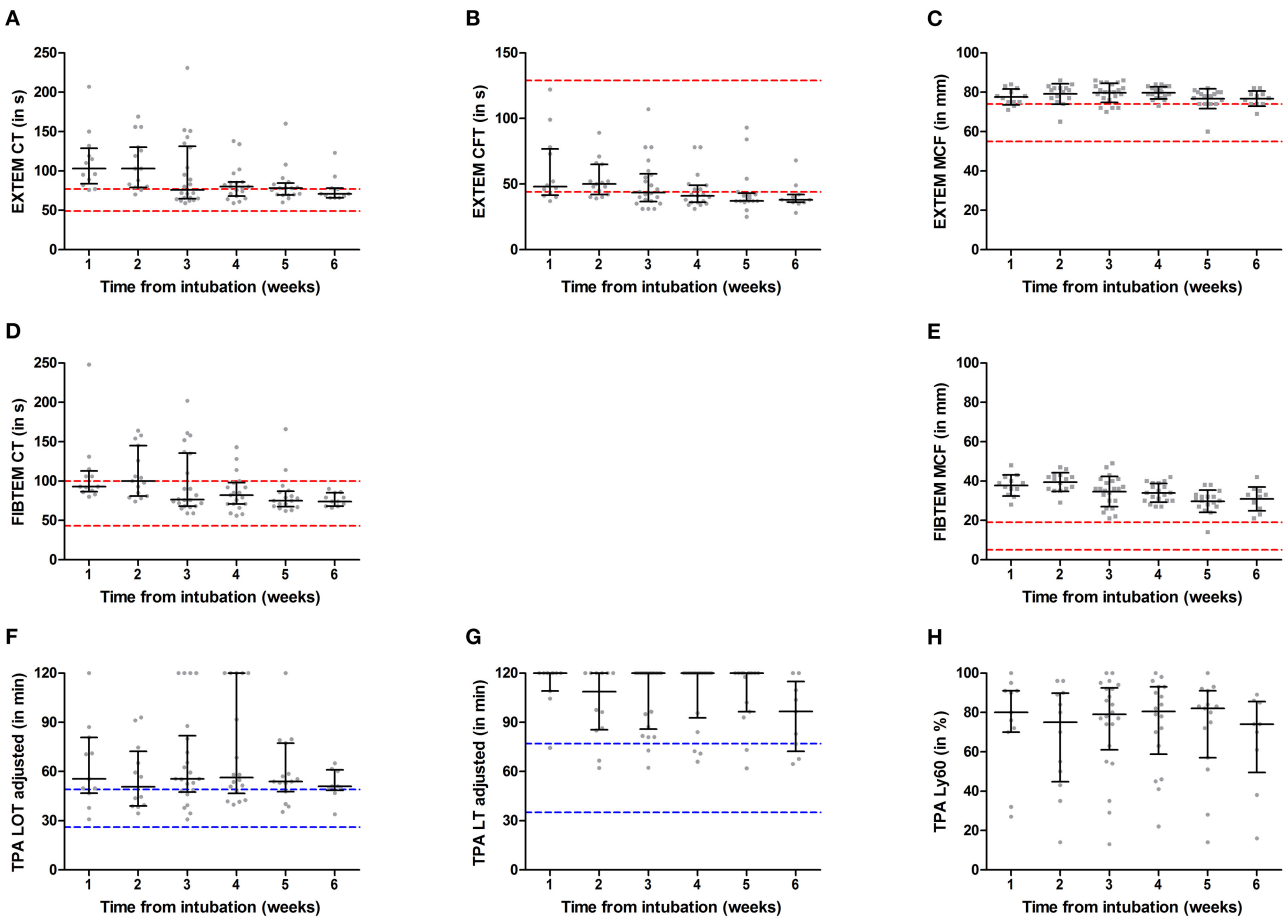
ROTEM EXTEM **(A–C)**, FIBTEM **(D,E)**, and tissue plasminogen activator (TPA) **(F–H)** measurements over 6 weeks from intubation in a critically ill COVID-19 population. Results are presented as mean with standard deviation (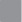) or median with interquartile range (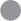); gray symbols illustrate individual measurements. Red dashed lines represent MUMC+ ROTEM reference ranges and blue dashed lines represent tPA ROTEM reference ranges as determined by Kuiper et al. (20). CT, clotting time; CFT, clot formation time; MCF, maximum clot firmness; LY60, lysis index at 60 min; LOT, lysis onset time; LT, lysis time.

The median [IQR] time between the day of intubation and first ROTEM measurement was 18 [6-25] days. We included a total of 36 patients in the hemostasis subcohort, who had a total number of 107 ROTEM measurements varying between 1 and 6 per patient. This led to a weekly total number of ROTEM measurements of 12 for week 1, 15 for week 2, 22 for week 3, 19 for week 4, 17 for week 5, 11 for week 6, and 11 for >week 6. In 97 samples (91%), tPA ROTEM was also performed. ROTEM results per week from intubation are presented in Table 2 and Figure 2.

EXTEM CT was largely prolonged during the first 2 weeks after intubation in COVID-19 ICU patients, as illustrated in Figure 2A. However, over time median and IQR decreased toward the reference range. Forty measurements (37.4%) with normal PT presented with prolonged EXTEM CT. Though the number of prolonged CT values remains consistently higher than PT, Table 4 illustrates that both tests classify fewer patients as prolonged as weeks from intubation increase.

**Table 4:** Number (percentage) of prolonged EXTEM clotting time (CT) and prothrombin time (PT) measurements.

**Week number**	**1**	**2**	**3**	**4**	**5**	**6**	**>6**	**Total (all weeks)**
Prolonged EXTEM CT	10 (83.3%)	13 (86.7%)	11 (50.0%)	11 (57.9%)	9 (52.9%)	3 (27.3%)	1 (10%)	58 (54.2%)
Prolonged PT	4 (33.3%)	2 (13.3%)	6 (27.3%)	5 (26.3%)	2 (11.8%)	0 (0%)	0 (0%)	19 (17.8%)

**Figure 3:**
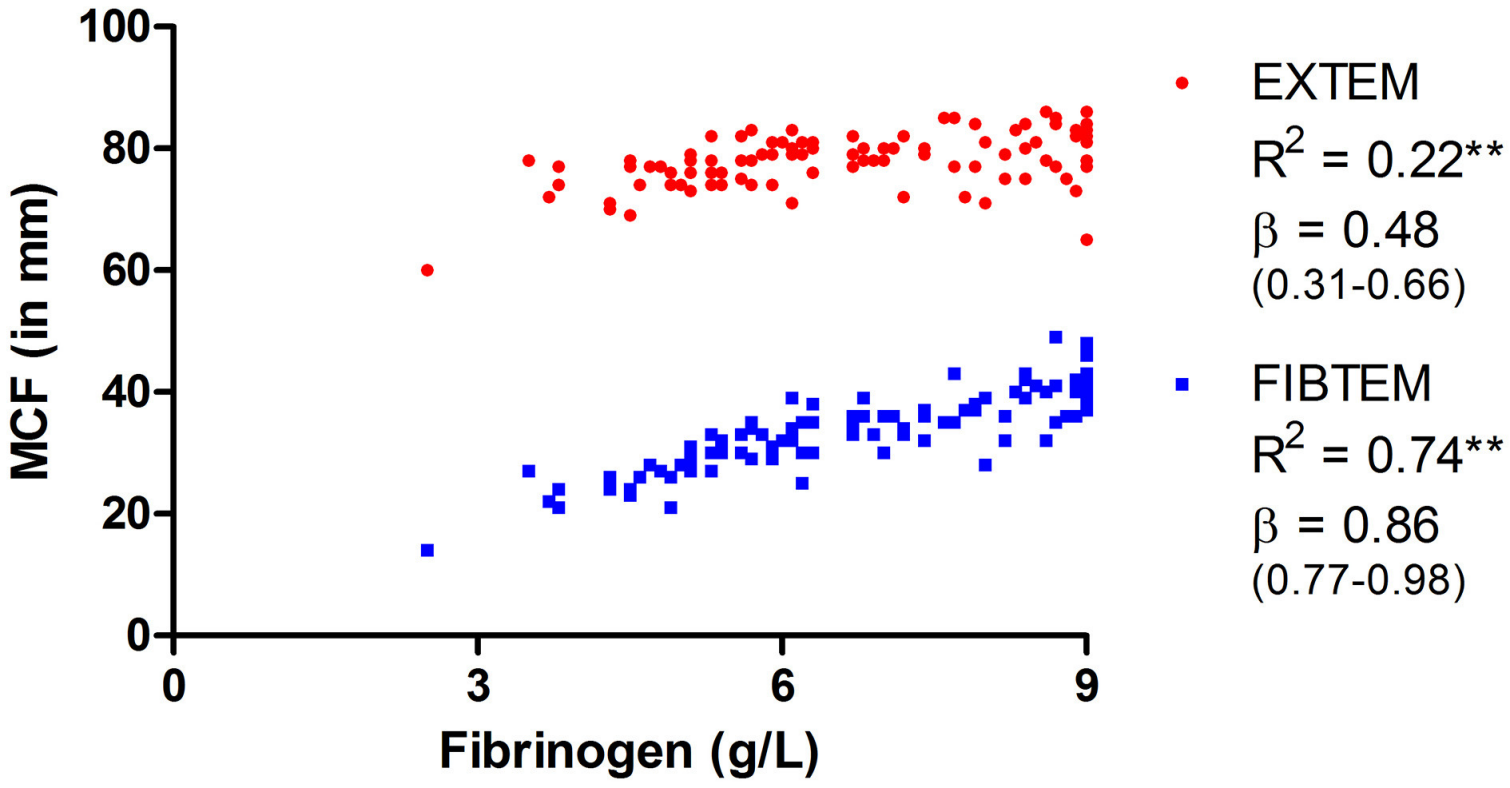
Linear regression between fibrinogen and maximum clot firmness (MCF) of EXTEM and FIBTEM. Presented are *R*^2^ and standardized β coefficient (95% CI). **p < 0.001.

The results show an increase in MCF for both EXTEM and FIBTEM assays, which persisted over 6 weeks, as illustrated with medians above the reference range's upper limit in Figures 2C,E. This hypercoagulability was considered more evident in FIBTEM compared to EXTEM as median values (and IQR) exceeded the reference range more prominently. Respectively, 81 and 99% of all EXTEM and FIBTEM MCF values were above the reference range. Linear regression analyses (Figure 3) showed that fibrinogen was more positively associated with FIBTEM MCF [β (95% CI) = 0.86 (0.77–0.98)] as compared to EXTEM MCF [β (95% CI) = 0.48 (0.31–0.66)]. Additionally, EXTEM MCF was positively associated with platelet count [*R*^2^ = 0.55, β (95% CI) = 0.74 (0.62–0.87), *p*-value < 0.001].

TPA ROTEM fibrinolysis parameters, presented in Figures 2F,G,H, illustrate initiation (LOT) and propagation (LT and Ly60) of clot breakdown. LOT and LT were unmeasurable within 2 h in, respectively, 11 and 56 samples, suggesting no or little clot breakdown occurred. Clot breakdown of 90% (LT) was not achieved in more than half of the samples.

A hypofibrinolytic phenotype was illustrated in all tPA ROTEM parameters, though most evident in LT. The median for LT in most weeks is consistent with the tPA measurements' cutoff value (120 min). Figure 2F further illustrates that the median of LOT is continuously above the reference range.

Data stratified by mortality are provided in Supplementary Tables 1, 2. Deceased patients had persisting higher D-dimer and prolonged aPTT values, reflecting the over-representation of ECMO and RRT in deceased patients. Additional sensitivity analyses excluding the six patients on ECMO during their stay in the ICU did not alter any of the conclusions.

## Discussion

Our serial and comprehensive study to characterize COVID-19-associated coagulopathy by (tPA) thromboelastometry has four main findings: (1) a persisting increase in MCF that was more prominent in FIBTEM compared to EXTEM, illustrating hypercoagulability that is at least partly fibrinogen dependent; (2) a prolonged EXTEM CT in 54% of all measurements; (3) a discordance between EXTEM CT and PT observed in 37% of the samples, possibly suggesting missing coagulopathy recognition in routine hemostatic assays; and (4) a persistently increased LOT and LT measured with tPA ROTEM, illustrating hypofibrinolysis. Overall, COVID-19-positive ICU patients presented with persisting hypercoagulability and hypofibrinolysis despite anticoagulant treatment.

Of note, patients received different, individualized anticoagulant treatment regimens. COVID-19 patients mainly received prophylactic LMWH, therapeutic LMWH, or (therapeutic) UFH, as illustrated in Table 3. The inclusion of all mechanically ventilated COVID-19 ICU patients, irrespective of ECMO and/or CRRT, not only makes for a heterogeneous group but also reflects the clinical reality of the COVID-19 ICU population. Sensitivity analyses excluding patients receiving ECMO during ICU admission did not alter any of the conclusions. Additionally, heparins execute limited effects on the ROTEM parameters; EXTEM and FIBTEM contain a heparin neutralizing agent (polybrene) and no clear fibrinolytic effect of heparins can be observed in tPA ROTEM (28). Taken together, the individualized treatment regimens, though illustrating the heterogeneous ICU population, do not in itself affect ROTEM results.

Although previous studies (15, 29–37) partly addressed these issues, measurements were either not serial, prospective, or did not assess both coagulation and fibrinolysis within a well-defined cohort (22) (Supplementary Figure 1). Hypercoagulability in COVID-19-positive ICU patients, illustrated by increased clot stiffness/amplitude in viscoelastic assays, has been extensively reported in the literature (15, 29–37). Routine hemostatic assays, such as PT, were often within reference ranges, highlighting the relevance of the addition of global hemostatic assays to identify coagulopathy in these patients. Only two studies performed viscoelastic testing in COVID-19 patients on multiple occasions, with a follow-up period of 10 and 14 days showing a significant decrease in clot stiffness over time and a significant decrease in FIBTEM MCF, respectively (29, 30). This decrease in clot stiffness (MCF) over time was not observed in our cohort. Notably, the decrease in fibrinogen over the first 2 weeks was more pronounced in both previous studies (29, 30), compared to our cohort, possibly explaining the observed lack of decrease in clot stiffness. The diagnostic accuracy of ROTEM to classify sepsis-associated coagulopathy has been studied and demonstrated an evident ability of EXTEM to discriminate patients with and without disseminated intravascular coagulation (DIC) (38). Though COVID-19-associated coagulopathy is distinct from DIC (14), these results suggest viscoelastic testing's applicability in identifying coagulopathy in critically ill patients.

Several studies report decreased fibrinolysis in viscoelastic tests (15, 34–36). COVID-19 patients present with paradoxical fibrinolytic findings; severely increased D-dimer on the one hand, but a hypofibrinolytic viscoelastic profile on the other. Additionally, both pro-fibrinolytic tPA and anti-fibrinolytic plasminogen activator inhibitor-1 (PAI1) are increased (37, 39). Assessment of fibrinolysis in routine viscoelastic assays remains limited since little fibrinolysis is likewise seen in patients and healthy controls. TPA-based ROTEM assays may provide a complete view of the fibrinolytic properties in these patients. Kuiper et al. (20) demonstrated a striking difference in ROTEM fibrinolysis parameters between healthy controls and septic patients, the latter presenting with a hypofibrinolytic profile. Nougier et al. (39) showed a similar hypofibrinolytic profile with tPA-activated ROTEM in a small cohort of COVID-19 ICU patients (*n* = 23), using a higher tPA concentration (0.625 μg/ml) as compared to our assay. A decreased fibrinolytic potential was associated with thromboembolic events in COVID-19 patients in other studies (15, 39). Though confirming a general hypofibrinolytic state persisting over several weeks, the extent of hypofibrinolysis remains somewhat elusive in our cohort since most patients presented with LT at cutoff value (120 min). A higher *in vitro* tPA concentration might be beneficial to differentiate the level of hypofibrinolysis in COVID-19 ICU patients. However, other ROTEM fibrinolysis markers (LOT and LY60) might be able to provide this differentiation in our current validated and standardized assay. We hypothesize that tPA ROTEM fibrinolysis parameters predict the occurrence of thromboembolic events in COVID-19 ICU patients, although further research analyzing clinical outcomes is required. In addition to tPA ROTEM, other fibrinolysis markers (e.g., α2-antiplasmin, tPA, and PAI-1) are of interest to unravel the pathophysiology underlying the observed hypofibrinolytic phenotype.

The major determinants of overall clot strength in viscoelastic testing are platelets and fibrinogen (40). The FIBTEM assay, in which platelets are inhibited by cytochalasin D, is mostly dependent on fibrinogen for clot formation. Increased FIBTEM MCF, as measured in COVID-19 patients, is likely caused by high fibrinogen levels. One might consider the hypercoagulability, as measured with EXTEM MCF above the reference range, to be dependent on fibrinogen as well. However, linear regression shows a large difference in the association of fibrinogen with FIBTEM compared to EXTEM.

Additionally, the decrease in fibrinogen and, consequently, FIBTEM MCF was not observed in EXTEM MCF. Together these data may suggest that platelet (activation) contributes to increased clot stiffness. Indeed, Manne et al. showed increased platelet activation and aggregation in COVID-19 ICU patients (41), and EXTEM MCF was positively associated with platelet count in our patients. Thus, although hyperfibrinogenemia is undoubtedly associated with increased clot stiffness, it is likely that other factors, including platelets, contribute to COVID-19 hypercoagulability.

An unexpected EXTEM CT prolongation (>77 s) was observed in 29 (81%) patients and 54% of all measurements. Considering the high doses of heparins administered (specifically UFH), the heparin inhibiting potential of polybrene in the EXTEM reagent might be exceeded. However, according to the manufacturer's instructions, the anti-Xa activity of 2–3 IU/ml should not result in a prolonged EXTEM CT. Since the highest measured anti-Xa level was 1.85 IU/ml, it is unlikely that heparins cause this prolongation. In addition, the EXTEM CT prolongation was not matched by PT. Although these assays have different reagents and measuring techniques, both represent the initiation of clot formation and use tissue factor as the activator. Previous studies in pediatric surgery patients and patients with various hemostatic disorders (including patients undergoing cardiac surgery) illustrated PT prolongations with normal EXTEM CT results (42, 43). Our results show a contrary course with a predominantly normal PT and a prolonged EXTEM CT, as shown in Table 4. Even though these assays both reflect the initiation of clot formation via the extrinsic pathway, the detection mechanism, reagent concentrations, and patient material (whole blood vs. plasma) used are completely different. These factors likely contribute to the observed discordance between the assays. Since the prolonged EXTEM CT is more in line with the observed COVID-19-associated coagulopathy compared to the predominantly normal PT results, we believe that ROTEM results may describe the hemostatic status in these patients more accurately.

Alternatively, a prolonged clotting time in COVID-19 patients could also be due to increased concentrations of D-dimer, fibrinogen, and, thus, fibrinogen degradation products (FDPs). As such, several animal studies have shown an inhibitory effect of FDPs on clotting assays (44, 45). However, the concentration of FDPs in these studies (0.05–2.5 g/L) largely exceeded our patients' expected concentration, deeming a causative role unlikely. Additionally, only a minimal association between D-dimer and EXTEM CT and PT was achieved (data not shown). All in all, we observe a prolonged EXTEM CT, which was unexplained.

Our study has some strengths and limitations. Strengths include the prospective and longitudinal nature of the study. To our knowledge, no serial assessment of tPA-based ROTEMs or follow-up after more than 2 weeks have been reported in the current literature, making this the first study to perform such comprehensive follow-up in a COVID-19 population. Furthermore, no previous study assessed both coagulation and fibrinolysis as thoroughly in these patients.

Limitations to our study mainly involve its generalizability and applicability in clinical practice. Due to the buildup of our cohort, not all patients were followed from ICU admission. As a result of this, we collected extensive follow-up over a long period, but conclusions concerning individual disease courses cannot be drawn from our data. The inclusion of all mechanically ventilated COVID-19 patients, irrespective of ECMO and CRRT, makes for a heterogeneous population, suggesting careful interpretation of the results and limited generalizability to other ICU populations. Additionally, because of the relatively small number of patients, no conclusions can be drawn regarding the risk for adverse outcome (VTE or mortality), which would have added to the study value. We focused on studying whether altered ROTEM parameters persisted or changed over time in severe COVID-19 infection. Therefore, we used descriptives of serial measurements. Our observations indicate that any altered ROTEM parameter studied during the course of severe COVID-19 infection can be used to investigate its associations with other coagulation markers and outcome, independent of the moment of measurement. This is important when pooling ROTEM data of several COVID-19 observational studies that used different sampling moments in time during the course of severe COVID-19 infection. Furthermore, beyond 6 weeks, insufficient measurements remained for analysis. However, the consistency of the results during the first 6 weeks of ICU admission suggests that little change is expected by extending this timeframe.

ROTEM analysis was not performed in all patients of the MaastrICCht cohort, as explained previously. Therefore, we compared the baseline characteristics of both the MaastrICCht and hemostasis subcohort to exclude selection bias (data not shown). Disease severity in the two cohorts is nearly identical, as indicated by similar SOFA and APACHE scores, and almost all other baseline characteristics are comparable except for comorbidities. Notably, within our study population, only a few comorbidities were present compared to the entire MaastrICCht cohort and other study populations. It is common practice in the Netherlands to initiate conservative (and/or palliative) care in COVID-19 patients with preexisting frailty or severe comorbidities. Therefore, only relatively healthy patients were transferred to the ICU for mechanical ventilation. A recent study in our center showed that, of all admitted COVID-19 patients, 35.2% had some form of treatment restrictions (46). This shows that COVID-19 related coagulopathy is not limited to patients with multiple comorbidities since our study sample with relatively healthy individuals presents with significant hemostatic abnormalities. This implies that the severity of the COVID-19-associated coagulopathy might be even more pronounced in other, more severely affected, ICU populations.

From a practical point of view, it must be acknowledged that tPA ROTEM measurement is labor-intensive and requires the “old” ROTEM delta device with manual pipetting, limiting its applicability to smaller hospitals. It might also not be feasible for huge patient volumes. Nonetheless, we did manage to set up and execute tPA ROTEM measurements during the first COVID-19 wave and expect that, with proper logistics, larger hospitals could still manage routine tPA ROTEM measurements for their ICU patients. Application of tPA ROTEM might aid in the characterization of COVID-19 and similar septic pathologies.

In conclusion, we found a persisting hypercoagulability and hypofibrinolysis, indicating severe and persisting COVID-19-associated coagulopathy as measured with (tPA) ROTEM. We hypothesize that hypercoagulability and hypofibrinolysis are associated with thromboembolic complications and aim to further study the association between ROTEM parameters, clinical variables, and outcomes in the MaastrICCht cohort. Future research should focus on applying ROTEM and other global hemostatic assays, such as thrombin generation, on identifying critically ill COVID-19 patients at risk for thrombosis. Comprehensive laboratory testing might help guide the optimal anticoagulant treatment strategy to ultimately improve morbidity and mortality in COVID-19 patients.

## Data Availability Statement

The datasets presented in this article are not readily available because no concrete agreements on data sharing have been made yet. Before any data is shared outside the MUMC+, a datasharing plan will be drawn up, in consultation with the data officer, that conforms to relevant laws and regulations concerning personal data. Requests to access the datasets should be directed to Anne-Marije Hulshof, annemarije.hulshof@mumc.nl.

## Ethics Statement

The studies involving human participants were reviewed and approved by Medisch Ethische Toetsingscomissie (METC) azM/UM, Maastricht UMC+, P. Debyelaan 25, 5800, 6202 AZ Maastricht azM/UM (2020-1565/ 300523). The patients/participants provided their informed consent to participate in this study.

## Author Contributions

A-MH and RB contributed to the design of the manuscript, performed statistical analysis, and were the main authors. MM collected clinical data. PV performed laboratory analysis. BB and IH developed the design of the MaastrICCht cohort. MM, TB, JS, RO, BS, AS, HS, PV, and IH critically reviewed the manuscript. BB, HT, and YH contributed to the concept and design of the manuscript, and revised the intellectual content. All authors contributed to the article and approved the submitted version.

## Conflict of Interest

RO has received research support an honoraria from Bayer, Pfizer/BMS, Leo Pharma, Portola, and Sanofi. HT has received grants from Bayer and Pfizen, is a consultant for Alveron, and a share holder of Coagulation Profile. YH reports that ROTEM reagents were previously provided for research and development. The remaining authors declare that the research was conducted in the absence of any commercial or financial relationships that could be construed as a potential conflict of interest.
