# Supplementary material for: Serial EXTEM, FIBTEM, and tPA Rotational Thromboelastometry Observations in the Maastricht Intensive Care COVID Cohort—Persistence of Hypercoagulability and Hypofibrinolysis Despite Anticoagulation
Source: Front Cardiovasc Med. 2021 Apr 26;8:654174. doi: 10.3389/fcvm.2021.654174 (PMC8107372; doi:10.3389/fcvm.2021.654174)
Supplement: Supplementary file 1 [file Data_Sheet_1.pdf]

Supplementary materials

Figure S.1 – Flowchart of patient inclusion and measurements

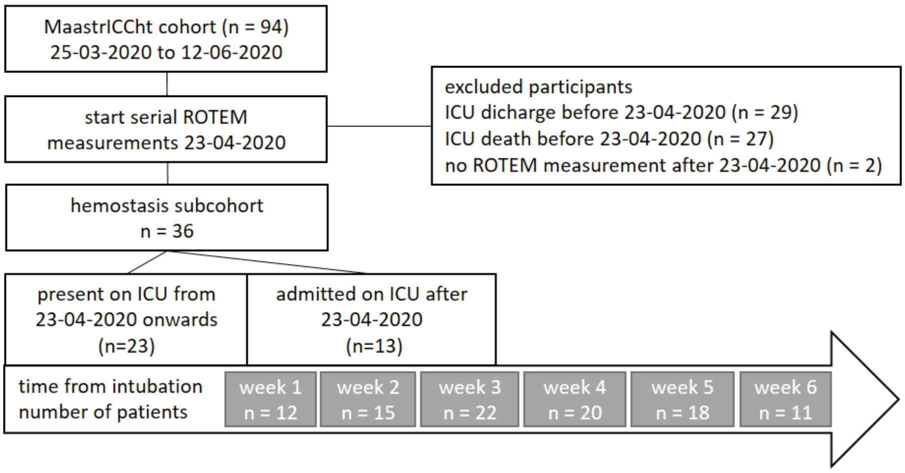

Legend: serial rotational elastometry (ROTEM) was timed from intubation (at our or transferring institution).
One missing ROTEM measurement in week 4 and week 5 explains difference in number of patients presented and Table 2; MaastrICCht, Maastricht Intensive Care Covid; ICU, Intensive Care Unit.

Table S.1 – baseline characteristics stratified by mortality

**Parameter**	**Mean (SD) or Median [IQR]**<br><br>**N (%)**	
**General**	Discharged (n=29)	Deceased (n=7)
Age (years)	61 [55-70]	61 [48-67]
Gender (male)	23 (79.3%)	6 (85.7%)
BMI (kg/m2)	27.8 [26.0-29.2]	26.9 [21.9-30.7]
**Medical history**		
Chronic kidney disease	1 (3.4%)	0 (0%)
Diabetes mellitus	1 (3.4%)	0 (0%)
Hypertension	9 (32.1%)*	1 (14.3%)
Malignancy	3 (10.3%)	1 (14.3%)
Myocardial infarction	1 (3.4%)	1 (14.3%)
Peripheral vascular disease	1 (3.4%)	0 (0%)
**Medication prior to admission**		
Antiplatelet agent		
• *Acetylsalicylic acid*	3 (10.3%)	1 {14.3%)
DOAC		
• *Apixaban*	1 (3.4%)	0 (0%)
• *Dabigatran*	1 (3.4%)	0 (0%)
• *Rivaroxaban*	1 (3.4%)	0 (0%)
**ICU admission**		
Admission origin		
• *Emergency department*	8 (27.6%)	1 (14.3%)
• *Regular ward*	14 (48.3%)	0 (0%)
• *Transfer from other ICU*	7 (24.1%)	6 (85.7%)
APACHE II at admission	15 [13-18]	15 [10-25]
SAPS II at admission	38 [29-43]	44 [31-48]
**ICU events**		

Length of stay ICU (days)	34 [22-42]	23 [19-46]
Time on ventilator (days)	27 [17-32]	23 [19-46]
CRRT during ICU stay (yes)	4 (13.8%)	6 (85.7%)
ECMO during ICU stay (yes)	3 (10.3%)	3 (42.9%)
CT confirmed Pulmonary embolism (yes)	17 (58.6%)	3 (42.9%)
Compression ultrasonography confirmed deep venous thrombosis (yes)	1 (3.4%)	0 (0%)

*data irretrievable for 1 patient

Table S.2 – SOFA score, routine laboratory, standard ROTEM and tPA ROTEM in COVID-19 patients per week from intubation stratified by mortality

**Parameter**	**Week number**	**1**	**2**	**3**	**4**
Routine laboratory					
SOFA-score	Discharged	7 [6-7]	7 [4-8]	5 [3-6]	7 [6-8]
	Deceased	7 [7-13]	8 [7-12]	11 [10-11]	6 [4-12]
Fibrinogen (g/L)	Discharged	8.9 [8.4-9]	9 [8.7-9]	7.2 [6.2-8.5]	6.3 [5.7-8.2]
	Deceased	8.2 [5.6-8.6]	8.4 [6.8-9]	7.8 [7.0-8.7]	5.1 [4.7-5.4]
D-dimer (ng/mL)	Discharged	1451 [965-3537]	2370 [1339-3985]	3102 [2271-4454]	1825 [1537-2680]
	Deceased	3951 [1625-30691]	5230 [2758-7557]	10637 [8108-12015]	8150 [1674-20166]
PT (s)	Discharged	10.9 [10.9-12.2]	11.4 [10.9-11.6]	11.4 [11.2-12.1]	11.6 [11.1-12.4]
	Deceased	13.2 [11.5-13.7]	11.7 [11.2-17.4]	11.8 [11.7-12.9]	11.7 [11.0-18.6]
aPTT (s)	Discharged	36 [31-37]	34 [30-38]	30 [28-40]	31 [29-33]
	Deceased	65 [32-65]	53 [41-55]	73 [65-75]	54 [26-77]
Platelet count (10^9/L)	Discharged	279 [244-357]	333 [237-531]	391 [329-590]	401 [345-608]
	Deceased	255 [167-378]	375 [261-384]	349 [170-464]	267 [200-335]
Standard ROTEM	**Week number**	**1**	**2**	**3**	**4**
Number of patients	Discharged	9	11	17	16
	Deceased	3	4	5	3
EXTEM CT (s)	Discharged	96 [89-119]	103 [78-130]	71 [64-89]	79 [70-86]
	Deceased	115 [76-207]	107 [84-141]	130 [104-135]	82 [65-138]
EXTEM CFT (s)	Discharged	47 [41-73]	50 [40-65]	40 [35-49]	39 [36-46]
	Deceased	49 [46-122]	50 [47-59]	60 [55-68]	50 [41-78]
EXTEM MCF (mm)	Discharged	78 [75-82]	81 [77-82]	80 [79-85]	81 [79-83]
	Deceased	75 [75-78]	81 [78-83]	78 [72-83]	77 [76-80]
FIBTEM CT (s)	Discharged	93 [88-80]	100 [81-126]	76 [68-90]	81 [69-95]

	Deceased	115 [83-248]	115 [82-155]	36 [30-37]	85 [71-143]
FIBTEM MCF (mm)	Discharged	39 [37-42]	41 [35-42]	36 [30-40]	34 [31-39]
	Deceased	33 [32-36]	40 [36-45]	36 [30-37]	30 [28-35]
TPA ROTEM	**Week number**	**1**	**2**	**3**	**4**
Number of patients	Discharged	9	9	17	15
	Deceased	3	3	5	3
TPA LY60 (%)	Discharged	78 [51-91]	55 [43-80]	80 [74-92]	84 [70-93]
	Deceased	91 [72-100]	90 [89-96]	77 [54-92]	46 [22-82]
TPA LOT (min)	Discharged	52.58 [43.77-75.62]	43.77 [38.78-56.77]	55.47 [48.23-79.95]	58.08 [50.72-120]
	Deceased	71.07 [46.72-120]	74.62 [65.15-91.15]	55.55 [39.45-71.63]	41.53 [39.70-58.00]
TPA LT (min)	Discharged	120 [106.73-120]	96.27 [85.10-120]	120 [96.47-120]	120 [95.65-120]
	Deceased	120 [120-120]	120 [120-120]	87.27 [81.70-120]	120 [65.90-120]

Week 5 and 6 were excluded from this analysis due to limited number of deceased patients measured, respectively, 2 and 1.
